# REGOSARC: Regorafenib versus placebo in doxorubicin‐refractory soft‐tissue sarcoma—A quality‐adjusted time without symptoms of progression or toxicity analysis

**DOI:** 10.1002/cncr.30661

**Published:** 2017-03-10

**Authors:** Vincent Berry, Laurent Basson, Emilie Bogart, Olivier Mir, Jean‐Yves Blay, Antoine Italiano, François Bertucci, Christine Chevreau, Stéphanie Clisant‐Delaine, Bernadette Liegl‐Antzager, Emmanuelle Tresch‐Bruneel, Jennifer Wallet, Sophie Taieb, Emilie Decoupigny, Axel Le Cesne, Thomas Brodowicz, Nicolas Penel

**Affiliations:** ^1^ Medical Oncology DepartmentOscar Lambret CenterLilleFrance; ^2^ Methodology and Clinical Research PlatformSIRIC OncoLilleLilleFrance; ^3^ Biostatisitics and Methodology UnitOscar Lambret CenterLilleFrance; ^4^ Medical Oncology DepartmentGustave RoussyVillejuifFrance; ^5^ Medical Oncology DepartmentLéon Bérard CenterLyonFrance; ^6^ Medical Oncology DepartmentBergonié InstituteBordeauxFrance; ^7^ Medical Oncology DepartmentPaoli‐Calmette InstituteMarseilleFrance; ^8^ Medical Oncology DepartmentUniversity Cancer Institute of Toulouse–OncopoleToulouseFrance; ^9^ Clinical Research UnitOscar Lambret CenterLilleFrance; ^10^ Institute of PathologyMedical University of GrazGrazAustria; ^11^ Radiology DepartmentOscar Lambret CenterLilleFrance; ^12^ General HospitalMedical University of ViennaViennaAustria

**Keywords:** metastatic soft‐tissue sarcoma, placebo, quality‐adjusted survival, quality‐adjusted time without symptoms of progression or toxicity (Q‐TWiST), regorafenib

## Abstract

**BACKGROUND:**

In a placebo‐controlled, randomized phase 2 trial (ClinicalTrials.gov identifier NCT01900743), regorafenib improved progression‐free survival (PFS) for patients with doxorubicin‐pretreated advanced nonadipocytic sarcoma. A quality‐adjusted time without symptoms of progression or toxicity (Q‐TWiST) post hoc exploratory analysis was applied to provide an integrated measure of its clinical benefit.

**METHODS:**

In the base‐case analysis, each patient's overall survival (OS) was partitioned into 3 mutually exclusive health states: the time with a grade 3 or 4 adverse event (TOX), the time without symptoms of disease or grade 3 or 4 toxicity from treatment, and the time after tumor progression or relapse. The time spent in each state was weighted with a health‐state utility associated with that state and was summed to calculate the Q‐TWiST. The stability of the base‐case analysis was explored with several sensitivity analyses.

**RESULTS:**

In nonadipocytic sarcoma, the PFS was (4.0 months [2.6‐5.5 months] with regorafenib vs 1.0 month [0.9‐1.8 months] with a placebo; hazard ratio, 0.36 [0.25‐0.53]; P < .0001); the OS was 13.4 months (8.6‐17.3 months) with regorafenib and 9.0 months (6.8‐12.5 months) with a placebo (hazard ratio, 0.67 [0.44‐1.02]). With the classic definition of TOX (including all grade 3 and 4 clinical adverse events), the Q‐TWiSTs were 8.0 months (7.0‐9.0 months) with regorafenib and 5.7 months (4.9‐6.4 months) with a placebo (*P* < .001).

**CONCLUSIONS:**

For patients with doxorubicin‐pretreated soft‐tissue sarcoma, regorafenib significantly improved quality‐adjusted survival in comparison with a placebo. ***Cancer* 2017;123:2294–2302.** © 2017 The Authors. *Cancer* published by Wiley Periodicals, Inc. on behalf of *American Cancer Society*. This is an open access article under the terms of the Creative Commons Attribution NonCommercial License, which permits use, distribution and reproduction in any medium, provided the original work is properly cited and is not used for commercial purposes.

## Abstract 2

In patients with doxorubicin‐pretreated nonadipocytic soft‐tissue sarcoma, regorafenib significantly improves quality‐adjusted survival in comparison with a placebo (8.0 vs 5.7 mo; *P* < .001).

See also pages 2200‐2.

## INTRODUCTION

Soft‐tissue sarcoma (STS) accounts for approximately 2% of all adult cancers.^1^ Approximately 5% of patients with STS present with metastases at diagnosis. Moreover, in cases of localized STS, despite large en bloc resection followed by adjuvant radiotherapy, a rate of relapse with metastases of approximately 40% is seen. At an advanced stage when it is not amenable to curative‐intent surgery, STS remains a difficult disease to treat. The recommended first‐line treatment for advanced STS in 2016 is still doxorubicin or doxorubicin and ifosfamide–based chemotherapy,^2^ which provides an overall survival (OS) of approximately 18 months.^3^ In the case of progression or intolerance of these major drugs, there are different approved options, including dacarbazine,^4^ trabectedin, and pazopanib.^2^ Doxorubicin‐refractory metastatic STS remains an unmet need. We have conducted an international, randomized, double‐blind, placebo‐controlled phase 2 trial assessing the activity and safety of regorafenib in doxorubicin‐refractory metastatic STS. Regorafenib significantly improved progression‐free survival (PFS) in 3 of the 4 cohorts: leiomyosarcoma, synovial sarcoma, and other nonadipocytic sarcomas. Regorafenib did not meet the primary objective for liposarcoma patients.^5^

The primary aims of palliative systemic treatment in this setting are as follows: 1) to alleviate symptoms related to disease progression; 2) to slow down the tumor growth; 3) to maintain or, if possible, improve the quality of life; and 4) to prolong OS with dignity and without major toxicity. However, data supporting the clinical benefit of palliative systemic treatment in advanced STS are sparse.^6^, ^7^, ^8^ In the current study, we analyzed the impact of regorafenib on quality of life with a modeling approach based on the quality‐adjusted time without symptoms of progression or toxicity (Q‐TWiST).^9^, ^10^ This method takes into account the OS, PFS, and time without intolerable toxicity (among grade 3/4 toxicities) caused by treatment in 2 arms (here regorafenib and placebo arms). At the end, this approach estimates a weighted sum of time spent without treatment‐related toxicity and without disease progression. However, because the definition of an intolerable toxicity is highly subjective, we interviewed 60 proxy patients to set up the threshold defining such toxicity.

## MATERIALS AND METHODS

### REGOSARC Study

REGOrafenib in SARComa (REGOSARC) was an international, randomized, double‐blind, placebo‐controlled phase 2 trial assessing the activity and safety of regorafenib in 4 cohorts of patients: liposarcoma, leiomyosarcoma, synovial sarcoma, and other nonadipocytic sarcomas.^5^ The stratification factors at randomization were the country (Austria vs France) and prior exposure to pazopanib. In both arms, treatment was administered until 1 of the following events occurred: confirmed progressive disease according to Response Evaluation Criteria in Solid Tumors (RECIST; version 1.1), unmanageable or unacceptable toxicity, a treatment delay of more than 21 days for any reason, concurrent serious illness, or a patient's refusal of treatment or an investigator's decision to stop it. Patients allocated to the placebo could receive regorafenib upon documented progression (crossover). The tumor assessment was performed at the baseline, every month during the first 3 months, and every 3 months after that. A blinded, central radiological review was conducted to reduce the bias. The primary endpoint was PFS according to RECIST (version 1.1) and after a blinded, central radiological reading. The study was approved by ethical and regulatory committees: the French ethics committee (French North‐West IV Ethics committee; date of approval, March 21, 2013), the Austrian ethics committee (Ethics committee of Vienna Medical University; No. 1376/2013], and the French and Austrian drug agencies (French Drug Agency; date of approval, March 8, 2013). All study procedures were conducted in accordance with the Harmonised Tripartite Guideline on Good Clinical Practice of the International Conference on Harmonisation. Signed informed consent was obtained from all study participants before registration. This study is registered with ClinicalTrials.gov (NCT01900743).^5^

### Q‐TWiST: Base‐Case Analysis

**Figure 1:**
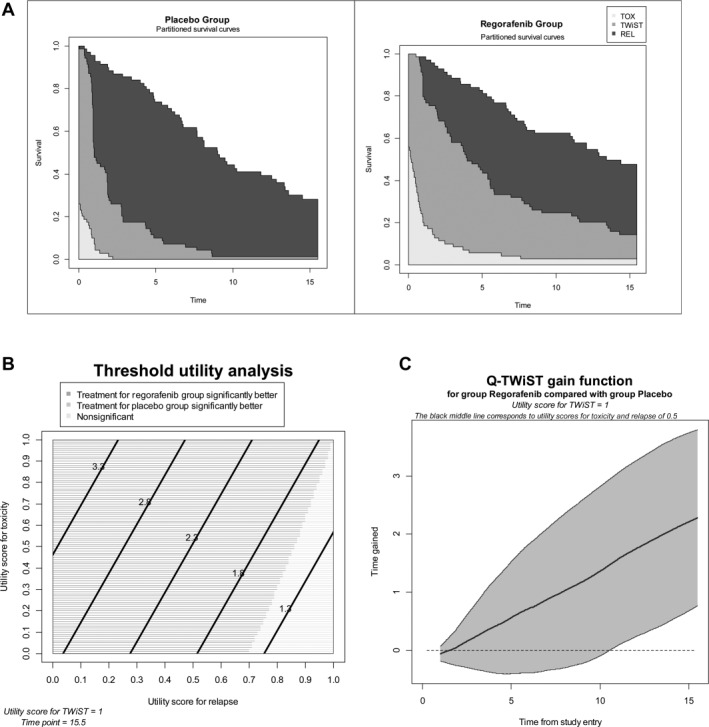
(A) Q‐TWiST: base‐case analysis of a placebo versus regorafenib. These graphs express the mean survival over time. The upper survival curves represent overall survival. The middle survival curves represent progression‐free survival. The lower survival curves represent survival without unacceptable treatment‐related toxicity. Between the overall survival curves and the progression‐free survival curves, the black represents the mean time spent between documented disease progression and death. Between the progression‐free survival curves and the curves of survival without unacceptable treatment‐related toxicity, the dark gray represents the Q‐TWiST. The light gray represents the mean time with unacceptable toxicity. (B) Threshold utility analysis. This graph depicts a utility analysis in which the utility of disease progression and the utility of treatment‐related toxicity vary from 0 to 1. The time horizon has been set at 15.5 months, and the utility of toxicity is constant (ie, 1). The horizontal axis represents the different values of disease progression from 0 to 1. The vertical axis represents the different values of treatment‐related toxicity. The dark gray represents a scenario in which regorafenib is superior to the placebo. On the contrary, the light gray represents a different scenario in which there is no significant difference between the placebo and regorafenib. There is no scenario in which the placebo is superior to regorafenib. (C) Difference in Q‐TWiST between the 2 treatment groups according to the time from randomization. This graph shows the difference in Q‐TWiST between the 2 arms over time and its 95% confidence interval. The horizontal axis represents the time spent from the date of randomization. The vertical axis represents the difference in Q‐TWiST between the 2 arms. This difference constantly increases over time, and this demonstrates that the gain in Q‐TWiST constantly increases with exposure to regorafenib. Q‐TWiST indicates quality‐adjusted time without symptoms of progression or toxicity; REL, time spent without disease progression; TOX, time with a grade 3 or 4 adverse event; TWiST, time spent without toxicity or disease progression.

The base‐case analysis was a post hoc analysis of REGOSARC trial data using the classic definition of unacceptable toxicity. After the randomization, the toxicity state or TOX was defined as the time spent with Common Terminology Criteria for Adverse Events grade 3 or 4 adverse events (AEs) before disease progression. In the current analysis, we have taken into account related and unrelated toxicity. The time spent with AEs was summed for each patient, and a day with multiple events was counted only once. TOX was determined as the time spent with clinical AEs before crossover, regardless of when the events occurred (biological AEs without clinical signs were excluded from the definition of TOX). However, any AEs that occurred after disease progression were excluded from the analysis. REL represents the time spent without disease progression according to RECIST (version 1.1) or death. TWiST represents the time spent without toxicity or disease progression (see Fig. 1A). The product‐limit method was used to estimate the mean amount of time in the following states: time with any toxicity after randomization but before progression (ie, TOX), time from randomization to progression or death (ie, PFS), and time from randomization until death from any cause (ie, OS). Survival curves that corresponded to TOX, PFS, and OS were plotted on the same graph for each treatment group. The areas between the curves represent the restricted mean durations of TWiST and REL as follows:

Duration of TWiST=Mean PFS−Mean time with toxicities

Duration of REL = Mean OS – Mean PFS

Q‐TWiST analysis uses these different periods of times, which are weighted by specific utilities. The mean Q‐TWiST for each treatment arm was calculated with the following formula:

Q−TWiST = (uTOX × TOX) + (uTWiST × TWiST) + (uREL × REL)

where uREL is the utility for the period without disease progression and is set to 0.5, uTOX is the utility for the period of symptoms and is set to 0.5, and uTWIST is the utility for the period without relapse and toxicity and is set to 1. Differences between treatment groups (regorafenib vs placebo) in the mean Q‐TWiST were calculated. Bootstrapping with 1000 iterations was performed to ensure accurate estimates of Q‐TWiST. The areas under the Q‐TWiST curves in both arms were measured and then compared.^9^, ^10^

We conducted 2 classic sensitivity analyses. The threshold sensitivity analysis explored different scenarios in which the utility of disease progression and the utility of treatment‐related toxicity varied from 0 to 1 (see Fig. 1B). The difference in Q‐TWiST between the 2 arms was measured over the time from the date of randomization (so‐called gain‐of‐function analysis; see Fig. 1C).^9^, ^10^

### Definition of Intolerable Toxicity

In the base‐case analysis and previously published analyses, the definition of TOX (ie, the time spent with Common Terminology Criteria for Adverse Events grade 3 or 4 AEs before disease progression) was arbitrary. It is likely that AEs differed in their impact on quality of life; patients' points of view were necessary to weigh these AEs. As a result, we interviewed 2 groups of patients, not included in the trial, to define an intolerable toxicity. Interviewing patients enrolled in the REGOSARC trial was not part of the study protocol, so we had to interview proxy patients. The first group of patients (the tyrosine kinase inhibitor [TKI] group) consisted of patients treated with a TKI, whatever the primary tumor was. The second group of patients (the STS group) consisted of STS patients receiving systemic treatment (mainly chemotherapy for metastatic disease). Patients assessed the tolerability of the main toxicities of regorafenib (alopecia, anorexia, asthenia, diarrhea, arterial hypertension, mucositis, and hand‐foot skin reactions) according to the grade (grades 2 and 3) and according to the duration of the toxicity. For each scenario, we used the event/grade/duration on a Likert‐scale hetero‐questionnaire with 4 values: acceptable, slightly unacceptable, unacceptable, and totally unacceptable. Every patient was interviewed by the same investigator (V.B.). The validity (reliability) of the questionnaire was assessed with Cronbach's α coefficient (good reliability was defined as α > .7). Comparisons of the points of view of the 2 groups (the STS and TKI groups) were analyzed with a principal component analysis.

### Q‐TWiST: Sensitivity Analysis Taking Into Account the Patients' Point of View

On the basis of the analysis of the hetero‐questionnaires, 6 alternative definitions of intolerable toxicity were used: 1) every toxicity regarded as at least slightly unacceptable by at least 20% of interviewed patients, 2) every toxicity regarded as at least slightly unacceptable by at least 33% of interviewed patients, 3) every toxicity regarded as at least slightly unacceptable by at least 50% of interviewed patients, 4) every toxicity regarded as unacceptable or totally unacceptable by at least 20% of interviewed patients, 5) every toxicity regarded as unacceptable or totally unacceptable by at least 33% of interviewed patients, and 6) every toxicity regarded as unacceptable or totally unacceptable by at least 50% of interviewed patients. Q‐TWiST was then recalculated with these 6 alternative definitions of TOX. In comparison with the base‐case analysis, the definitions of REL, TWiST, and utilities remained unchanged.

## RESULTS

### Definition of Intolerable Toxicity According to Interviewed Patients

From February 2015 to December 2015, 67 eligible patients were invited to participate; 7 patients refused. Thirty STS patients and 30 TKI patients were interviewed. The sex ratio was 2/1, and the mean age was 61 ± 12 years. Most of the TKI patients were treated with sunitinib (22 of 30).

The validity of the questionnaire was very good for the following toxicities (Cronbach's α coefficient > .7): anorexia (.92), asthenia (.87), diarrhea (0.92), arterial hypertension (.86), mucositis (.88), and hand‐foot skin reactions (.87). The validity was insufficient for alopecia (.59), and this particular AE was excluded from the analysis. The principal component analysis demonstrated that the assessments of toxicity tolerability were similar in the 2 groups of interviewed patients (data not shown). Table 1 summarizes the patients' point of view.

**Table 1:** Q‐TWiST: Base‐Case Analysis and Sensitivity Analysis

	Q‐TWiST, mo (95% CI)		Difference,	
Definition of TOX	Placebo	Regorafenib	mo (95% CI)	*P*
Base‐case analysis: every classic adverse event ≥ grade 3	5.68 (4.92‐6.45)	7.97 (6.96‐8.97)	2.28 (1.02‐3.55)	<.001
1. Every toxicity regarded as at least slightly unacceptable by at least 20% of interviewed patients	5.38 (4.65‐6.11)	6.52 (5.70‐7.33)	1.14 (0.04‐2.24)	.043
2. Every toxicity regarded as at least slightly unacceptable by at least 33% of interviewed patients	5.52 (4.80‐6.23)	6.81 (5.95‐7.69)	1.30 (0.19‐2.41)	.022
3. Every toxicity regarded as at least slightly unacceptable by at least 50% of interviewed patients	5.60 (4.85‐6.35)	7.27 (6.33‐8.21)	1.67 (0.46‐2.88)	.007
4. Every toxicity regarded as unacceptable or totally unacceptable by at least 20% of interviewed patients	5.58 (4.83‐6.34)	7.09 (6.18‐8.0)	1.51 (0.33‐2.70)	.012
5. Every toxicity regarded as unacceptable or totally unacceptable by at least 33% of interviewed patients	5.71 (4.95‐6.47)	7.49 (6.59‐8.40)	1.79 (0.58‐2.99)	.004
6. Every toxicity regarded as unacceptable or totally unacceptable by at least 50% of interviewed patients	5.74 (4.99‐6.49)	7.85 (6.86‐8.84)	2.11 (0.87‐3.35)	.001

Abbreviations: CI, confidence interval; Q‐TWiST, quality‐adjusted time without symptoms of progression or toxicity; TOX, time with a grade 3 or 4 adverse event.

### REGOSARC Trial Results

The primary analysis of this trial has already been reported elsewhere. Briefly, from August 2013 to November 2014, 182 patients were randomized across the 4 cohorts and included in the final intent‐to‐treat analysis. At the cutoff date (January 7, 2016), the number of required events was reached for the 4 cohorts. The trial did not meet the primary endpoint of superiority for liposarcoma patients. In nonadipocytic patients, regorafenib was associated with a statistically and clinically significant improvement in PFS (4.0 months [2.6‐5.5 months] with regorafenib vs 1.0 month [0.9‐1.8 months] with a placebo; hazard ratio, 0.36 [0.25‐0.53]; *P* < .0001). A trend toward an improvement in OS was observed despite the high number of patients who switched to regorafenib in the placebo arm (13.4 months [8.6‐17.3 months] with regorafenib vs 9.0 months [6.8‐12.5 months] with a placebo; hazard ratio, 0.67 [0.44‐1.01]; *P* = .06).

Before crossover, the most common clinically significant grade 3 or higher AEs were arterial hypertension (18% in the regorafenib arm vs 2% in the placebo arm), hand‐foot skin reactions (15% vs 0%), asthenia (13% vs 7%), diarrhea (4% vs 2%), mucositis (4% vs 0%), and anorexia (3% vs 4%). Table 1 suggests larger differences in TOX between the placebo and regorafenib arms for toxicities deemed to be intolerable by a larger proportion of patients.

### Q‐TWiST: Base‐Case Analysis

The Q‐TWiST is illustrated in Figure 1A. The TWiST was 2.01 months (95% confidence interval [CI], 1.43‐2.58 months) and 4.80 months (95% CI, 3.77‐6.00 months) in the placebo and regorafenib arms, respectively. The TOX was 0.22 months (95% CI, 0.11‐0.33 months) and 1.14 months (95% CI, 0.49‐1.78 months) in the placebo and regorafenib arms, respectively. The Q‐TWiST was 5.68 months (95% CI, 4.92‐6.45 months) and 7.97 months (95% CI, 6.96‐8.97 months) in the placebo and regorafenib arms, respectively (Table 1). The difference was significant (*P* < .0001) with an absolute Q‐TWiST gain of 2.28 months (95% CI, 1.02‐3.55). The threshold utility analysis showed that most of the utility values were associated with a significant improvement in Q‐TWiST with regorafenib (Fig. 1B). The Q‐TWiST gain of function according to time showed that the benefit constantly increased with the duration of treatment (Fig. 1C).

### Sensitivity Analysis Taking Into Account the Point of View of Interviewed Patients

Table 2 summarizes these analyses. Whatever the threshold defining TOX was, regorafenib was always associated with a significant improvement in Q‐TWiST.

**Table 2:** Tolerability Assessment of Toxicity by Interviewed Patients

AE	Acceptable, No. (%)	Slightly Unacceptable, No. (%)	Unacceptable, No. (%)	Totally Unacceptable, No. (%)
Alopecia				
Limited	38 (63)	20 (33)	2 (3)	0 (0)
Important	20 (33)	27 (45)	9 (15)	4 (7)
Anorexia				
Habitual nutrition				
1‐7 d/mo	52 (88)	3 (5)	4 (7)	0 (0)
8‐14 d/mo	46 (78)	9 (15)	3 (5)	1 (2)
>15 d/mo	30 (51)	19 (32)	9 (15)	1 (2)
Decreased nutrition				
1‐7 d/mo	52 (87)	4 (7)	3 (5)	1 (2)
8‐14 d/mo	43 (72)	12 (20)	4 (7)	1 (2)
>15 d/mo	28 (47)	18 (30)	12 (20)	2 (3)
Decreased nutrition + weight loss				
1‐7 d/mo	38 (63)	12 (20)	6 (10)	4 (7)
8‐14 d/mo	20 (33)	25 (42)	11 (18)	4 (7)
>15 d/mo	10 (17)	20 (33)	21 (35)	9 (15)
Hospitalization				
1‐7 d/mo	23 (38)	18 (30)	15 (25)	4 (7)
8‐14 d/mo	11 (18)	17 (28)	24 (40)	8 (13)
>15 d/mo	5 (8)	7 (12)	31 (52)	17 (28)
Diarrhea				
Feces increase 1‐3 times/d				
1‐7 d/mo	40 (67)	18 (30)	2 (3)	0 (0)
8‐14 d/mo	30 (50)	18 (30)	12 (20)	0 (0)
>15 d/mo	21 (35)	12 (20)	26 (43)	1 (2)
Feces increase 4‐6 times/d				
1‐7 d/mo	23 (38)	15 (25)	17 (28)	5 (8)
8‐14 d/mo	7 (12)	24 (41)	18 (31)	10 (17)
>15 d/mo	6 (10)	10 (17)	26 (44)	17 (29)
Feces increase ≥ 7 times/d				
1‐7 d/mo	6 (10)	17 (29)	17 (29)	19 (32)
8‐14 d/mo	3 (5)	6 (10)	25 (42)	25 (42)
>15 d/mo	3 (5)	5 (8)	24 (41)	27 (46)
Incontinence				
1‐7 d/mo	6 (10)	16 (27)	21 (36)	16 (27)
8‐14 d/mo	5 (8)	3 (5)	29 (49)	22 (37)
>15 d/mo	4 (7)	3 (5)	25 (42)	27 (46)
Hospitalization				
1‐7 d/mo	27 (46)	18 (31)	8 (14)	6 (10)
8‐14 d/mo	10 (17)	15 (25)	22 (37)	12 (20)
>15 d/mo	5 (8)	5 (8)	29 (49)	20 (34)
Mucositis				
Slightly painful				
1‐7 d/mo	51 (85)	7 (12)	2 (3)	0 (0)
8‐14 d/mo	44 (75)	9 (15)	5 (8)	1 (2)
>15 d/mo	36 (61)	11 (19)	9 (15)	3 (5)
Moderately painful				
1‐7 d/mo	39 (66)	13 (22)	6 (10)	1 (2)
8‐14 d/mo	24 (41)	17 (29)	16 (27)	2 (3)
>15 d/mo	18 (31)	11 (19)	23 (39)	7 (12)
Painful				
1‐7 d/mo	13 (22)	16 (27)	17 (29)	13 (22)
8‐14 d/mo	5 (8)	5 (8)	28 (47)	21 (36)
>15 d/mo	4 (7)	1 (2)	29 (49)	25 (42)
Hospitalization				
1‐7 d/mo	32 (55)	10 (17)	11 (19)	5 (9)
8‐14 d/mo	9 (16)	15 (26)	24 (41)	10 (17)
>15 d/mo	7 (12)	2 (3)	34 (59)	15 (26)
Hand‐foot skin reaction				
Slightly painful				
1‐7 d/mo	51 (86)	6 (10)	1 (2)	1 (2)
8‐14 d/mo	44 (75)	10 (17)	4 (7)	1 (2)
>15 d/mo	38 (64)	11 (19)	7 (12)	3 (5)
Moderately painful				
1‐7 d/mo	28 (47)	20 (34)	6 (10)	5 (8)
8‐14 d/mo	9 (15)	23 (39)	15 (25)	12 (20)
≥15 d/mo	4 (7)	11 (19)	28 (47)	16 (27)
Painful				
1‐7 d/mo	7 (12)	10 (17)	23 (39)	19 (32)
8‐14 d/mo	2 (3)	2 (3)	31 (53)	24 (41)
≥15 d/mo	2 (3)	1 (2)	31 (53)	25 (42)
Hospitalization				
1‐7 d/mo	30 (51)	14 (24)	7 (12)	8 (14)
8‐14 d/mo	11 (19)	15 (25)	19 (32)	14 (24)
≥15 d/mo	6 (10)	7 (12)	27 (46)	19 (32)
Arterial hypertension				
Without treatment				
1‐7 d/mo	56 (93)	2 (3)	2 (3)	0 (0)
8‐14 d/mo	55 (92)	2 (3)	3 (5)	0 (0)
≥15 d/mo	53 (88)	4 (7)	3 (5)	0 (0)
With 1 more treatment/d				
1‐7 d/mo	52 (87)	4 (7)	4 (7)	0 (0)
8‐14 d/mo	51 (85)	5 (8)	4 (7)	0 (0)
≥15 d/mo	49 (82)	7 (12)	4 (7)	0 (0)
With 2 or 3 more treatments/d				
1‐7 d/mo	38 (63)	12 (20)	9 (15)	1 (2)
8‐14 d/mo	32 (53)	15 (25)	11 (18)	2 (3)
≥15 d/mo	30 (50)	16 (27)	11 (18)	3 (5)
Hospitalization				
1‐7 d/mo	35 (59)	9 (15)	11 (19)	4 (7)
8‐14 d/mo	14 (24)	11 (19)	23 (39)	11 (19)
≥15 d/mo	8 (14)	7 (12)	29 (49)	15 (25)
Asthenia				
Without modification of activities				
1‐7 d/mo	43 (93)	2 (4)	1 (2)	0 (0)
8‐14 d/mo	40 (87)	3 (7)	2 (4)	1 (2)
≥15 d/mo	34 (74)	5 (11)	5 (11)	2 (4)
Need rest at least half‐d/d				
1‐7 d/mo	38 (83)	6 (13)	2 (4)	0 (0)
8‐14 d/mo	29 (63)	13 (28)	3 (7)	1 (2)
≥15 d/mo	19 (41)	15 (33)	8 (17)	4 (9)
Need rest more than half‐d/d				
1‐7 d/mo	25 (54)	10 (22)	7 (15)	4 (9)
8‐14 d/mo	9 (20)	20 (43)	10 (22)	7 (15)
≥15 d/mo	4 (9)	10 (22)	19 (41)	13 (28)
Hospitalization				
1‐7 d/mo	27 (59)	8 (17)	7 (15)	4 (9)
8‐14 d/mo	8 (17)	17 (37)	11 (24)	10 (22)
≥15 d/mo	5 (11)	3 (7)	23 (50)	15 (33)

## DISCUSSION

The current study shows that the 2 groups of interviewed patients similarly assessed the tolerability of 6 major AEs. Whatever threshold was used for defining intolerable toxicity, in comparison with a placebo, regorafenib significantly improved the Q‐TWiST by approximately 1.6 months. Despite the poor outcomes associated with doxorubicin‐refractory advanced STS and the occurrence of AEs related to regorafenib, this Q‐TWiST benefit appears statistically and clinically significant.

In the palliative‐care setting, the integration of quality of life into decision making and drug development is critical. Symptom burden is a major issue for patients with advanced sarcoma. Gough et al^8^ retrospectively assessed the symptom burden in 81 patients with advanced sarcoma; the median number of symptoms was 2 (range, 0‐5) before first‐line chemotherapy (n = 50) and 3 (range, 1‐6) at the time of the exclusive palliative‐care decision (n = 48). The commonest symptoms were pain and dyspnea. This justifies early supportive/palliative‐care interventions. The data assessing the quality of life of patients with advanced STS are sparse.^5^, ^6^, ^7^, ^8^, ^11^ The evidence demonstrating that systemic treatment decreases the symptom burden in patients with advanced sarcoma is missing. In the Pazopanib Explored in Soft Tissue Sarcoma (PALETTE) trial (pazopanib vs placebo), quality of life was assessed with a validated health‐related self‐questionnaire such as the Quality of Life Questionnaire Core 30.^7^, ^11^ In general, the health‐related quality of life declined over time over a similar range in both arms. Pazopanib was associated with a significant detrimental impact on the quality of life because of diarrhea, anorexia, nausea/vomiting, and asthenia.^7^, ^11^ Pazopanib treatment did not significantly reduce the symptom burden. In the current study, we used an alternative approach, that is, the Q‐TWiST methodology, to examine all dimensions: OS, PFS, and time without symptoms. Whatever method is used, we think that an assessment of the quality of life is necessary when the activity/safety of new options is being evaluated in the palliative setting.

To our knowledge, there was no prior study of STS patients and patients receiving regorafenib using Q‐TWiST analysis. Q‐TWiST analysis remains rarely used. This approach was initially used for patients with breast cancer receiving adjuvant chemotherapy. Q‐TWiST has been more recently used in the metastatic setting of different advanced solid tumors. We found 2 articles assessing Q‐TWiST in renal cell carcinoma patients receiving oral TKIs. Patil et al^9^ compared Q‐TWiSTs for patients receiving sunitinib and patients receiving interferon‐α. TOX was defined as every clinically significant grade 3 AE. Sunitinib was associated with a longer Q‐TWiST (5.8 vs 1.8 months), mainly because of significant improvements in PFS (+6 months). Beaumont et al^10^ compared the clinical benefits of pazopanib and sunitinib. TOX was defined as every grade 3 AE in their first approach and as every grade 2 AE in their second approach. There was no significant difference in Q‐TWiSTs with either of the approaches because PFS was similar in the 2 arms, but pazopanib was associated with a higher rate of AEs.

In the current study, we explored the impact of different definitions of TOX in a Q‐TWiST analysis. We used classic values of utilities (uTWiST = 1, uTOX = 0.5, and uREL = 0.5). These values are largely used in the literature, and we conducted sensitivity analyses demonstrating the stability of the results (Fig. 1B). The choice of utility values has already been explored in prior studies. Guest et al^12^ proposed different values of utilities: 0.30 in the case of disease progression, 0.43 in the case of stable disease, 0.51 in the case of a partial response, and 0.60 in the case of a complete response after palliative chemotherapy. However, that study did not take into account toxicity. Shingler et al^13^ proposed different utilities in the case of stable disease: 0.74 in the absence of toxicity, 0.50 in the case of pain, 0.49 in the case of dyspnea, 0.47 in the case of asthenia, 0.41 in the case of diarrhea, and 0.38 in the case of nausea/vomiting. In these 2 last studies, utilities were defined after interviews of subjects from the general population and not cancer patients. Reichardt et al^6^ defined some utilities in STS and osseous sarcoma patients experiencing stable disease during first‐line treatment (*u* 0.72) where *u* is utility, stable disease during second‐line treatment (*u* = 0.64), stable disease during third‐line treatment (*u* = 0.77), stable disease during a drug holiday (*u* = 0.77), and disease progression (*u* = 0.56). In this last study, toxicity utilities were not explored.

The current study has some limitations. First, we were not able to interview patients enrolled in the REGOSARC trial because this study was an international multicenter study with 30 enrolling sites. The current study required standardization and harmonization of patients' interviews; every patient had to be interviewed by the same investigator. As a result, we enrolled proxy patients affected by advanced STS or being treated with TKIs. The principal component analysis showed the convergence of the points of view of both groups of patients. Second, we focused the hetero‐questionnaire on 6 clinically relevant major AEs, and we did not integrate other AEs, such as myalgia, voice changes, or dyspnea. As a result, we might hypothesize that Q‐TWiST was slightly overestimated because the list of AEs entered into the model was not exhaustive. Some autoquestionnaires such as the patient‐reported outcomes version of the Common Terminology Criteria for Adverse Events could be useful for better and more objectively defining intolerable toxicity from the patients' perspective.

In the end, the presented results support the conclusion that compared with a placebo, regorafenib offers improved clinical and quality‐of‐life outcomes for patients with advanced/metastatic STS.

## FUNDING SUPPORT

The REGOSARC trial (ClinicalTrials.gov identifier NCT01900743) was fully funded by Bayer HealthCare SA; however, the current study was conducted by the study coordinators (Nicolas Penel and Thomas Brodowicz), their sponsor (the Oscar Lambret Cancer Center), and their statisticians. Bayer HealthCare SA had no role in the collection, analysis, or interpretation of data. The corresponding author had full access to all of the data and the final responsibility for submitting the manuscript for publication.

## CONFLICT OF INTEREST DISCLOSURES

Nicolas Penel, Thomas Brodowicz, and Jean‐Yves Blay received research funding from Bayer HealthCare SA. Olivier Mir reports personal fees from Lilly, Roche, Pfizer, Novartis, Bayer, Amgen, Astra‐Zeneca, and Bristol‐Myers Squibb outside the submitted work. Jean‐Yves Blay reports grants from Bayer, Novartis, and GlaxoSmithKline outside the submitted work. Christine Chevreau reports grants from Pfizer, Novartis, and Bristol‐Myers Squibb outside the submitted work. Axel Le Cesne declares personal fees from Novartis, PharmaMar, Pfizer, Lilly, and Ariad outside the submitted work. Thomas Brodowicz reports personal fees from Roche, Amgen, Bayer, Novartis‐GlaxoSmithKline, PharmaMar, Eli Lilly, and Eisai outside the submitted work.

## AUTHOR CONTRIBUTIONS

**Vincent Berry**: Study design, writing of the manuscript, and final approval of the manuscript. **Laurent Basson**: Study design, data analysis, writing of the manuscript, and final approval of the manuscript. **Emilie Bogart**: Study design, data analysis, writing of the manuscript, and final approval of the manuscript. **Olivier Mir**: Data collection, writing of the manuscript, and final approval of the manuscript. **Jean‐Yves Blay**: Data collection, writing of the manuscript, and final approval of the manuscript. **Antoine Italiano**: Data collection, writing of the manuscript, and final approval of the manuscript. **François Bertucci**: Data collection, writing of the manuscript, and final approval of the manuscript. **Christine Chevreau**: Data collection, writing of the manuscript, and final approval of the manuscript. **Stéphanie Clisant‐Delaine**: Writing of the manuscript and final approval of the manuscript. **Bernadette Liegl‐Antzager**: Data collection, writing of the manuscript, and final approval of the manuscript. **Emmanuelle Tresch‐Bruneel**: Study design, data analysis, writing of the manuscript, and final approval of the manuscript. **Jennifer Wallet**: Writing of the manuscript and final approval of the manuscript. **Sophie Taieb**: Data collection, writing of the manuscript, and final approval of the manuscript. **Emilie Decoupigny**: Writing of the manuscript and final approval of the manuscript. **Axel Le Cesne**: Data collection, writing of the manuscript, and final approval of the manuscript. **Thomas Brodowicz**: Data collection, writing of the manuscript, and final approval of the manuscript. **Nicolas Penel**: Study design, data collection, data analysis, writing of the manuscript, and final approval of the manuscript.
